# Artificial intelligence-assisted early screening of acute promyelocytic leukaemia in blood smears: a prospective evaluation of MC-100i

**DOI:** 10.3389/fonc.2025.1572838

**Published:** 2025-04-07

**Authors:** Fan Zhang, Pingjuan Liu, Jieyu Zhan, Jing Cheng, Hongxia Tan, Jiahang Zhang, Meiqi Song, Fengying Wu, Qiuyi Lin, Zhuangbiao Shi, Chanjun Yang, Meinan Wang, Qiu Li, Yang Wang, Liubing Li, Junxun Li

**Affiliations:** ^1^ Department of Medical Laboratory, First Affiliated Hospital of Sun Yat-sen University, Guangzhou, China; ^2^ Department of Pediatric, Baiyun District Maternal and Child Healthcare Centre, Guangzhou, China; ^3^ School of Medical Technology, Guangdong Medical University, Dongguan, China; ^4^ Yunkang School of Medicine and Health, Nanfang College, Guangzhou, China; ^5^ Department of Blood Transfusion, The Second Affiliated Hospital of Shantou University Medical College, Shantou, China; ^6^ IVD Domestic Clinical Application Department, Mindray Biomedical Electronics Co., Ltd., Shenzhen, Guangdong, China; ^7^ School of Laboratory Medicine and Biotechnology, Southern Medical University, Guangzhou, China

**Keywords:** acute promyelocytic leukaemia, MC-100i, early screening, artificial intelligence, morphology, blood smears, automated digital blood cell morphology analyser

## Abstract

**Objectives:**

Identification of abnormal promyelocytes is crucial for early diagnosis of Acute promyelocytic leukaemia (APL) and for reducing the early mortality rate of APL patients, which can be achieved by microscopic blood smear observation. However, microscopic observation has shortcomings, including interobserver variability and training difficulty. This is the first study evaluating the performance of MC-100i, an artificial intelligence (AI)-based digital morphology analyser, in identifying abnormal promyelocytes in blood smears and thus assisting in the early screening of APL.

**Methods:**

One hundred ninety-two patients suspected of having APL were enrolled prospectively. The precision, accuracy, consistency with manual classification and turnaround time of MC-100i were studied in detail.

**Results:**

The precision of MC-100i in identifying all cell types was acceptable. MC-100i had excellent performance in preclassifying normal cell types, but its sensitivities for identifying blasts, abnormal promyelocytes, promyelocytes and neutrophilic myelocytes were relatively low, respectively. The Passing-Bablok and Bland-Altman tests revealed that the preclassification abnormal promyelocyte percentage obtained with MC-100i was proportionally different from that obtained with manual classification, whereas the postclassification and manual classification results were consistent. The clinical sensitivity and specificity for the early screening of APL were 95.8% and 100.0%, respectively. The turnaround and classification times were significantly shorter with the use of MC-100i for both the technologist and the experienced expert.

**Conclusions:**

MC-100i is an effective tool for identifying abnormal promyelocytes in blood smears and assisting in the early screening of APL. It is useful when experienced morphological experts or advanced tests are not available.

## 1 Introduction

Acute promyelocytic leukaemia (APL) is a specific type of acute myeloid leukaemia (AML) characterised by t(15;17) translocation, promyelocytic leukaemia gene‐retinoic acid receptor alpha (*PML*::*RARA*) fusion oncoprotein transcripts and abnormal promyelocyte predomination (1, 2). APL accounts for approximately 5%-10% of AML cases (1, 3). Compared to other AML subtypes, APL has unique clinical urgency due to its rapid progression to fatal coagulopathy. The early mortality rate of APL is high (ranging from 5% to 29% in the literature) because patients tend to develop fatal haemorrhage and disseminated intravascular coagulopathy (4–6). However, APL is curable if treatment is administered immediately (2). These treatments include all‐trans retinoic acid (ATRA) and arsenic trioxide (ATO), both of which can reduce the risk of fatal bleeding and cure most APL patients (2, 3). Therefore, rapid and early screening is crucial to reduce the early death rate in APL patients (7).

Currently, the diagnosis of APL mainly involves microscopic morphological assessment of the bone marrow smears, flow cytometry and cytogenetic tests (1, 8, 9). However, these tests are invasive, expensive, time-consuming and resource-demanding (3, 7). A complete blood count (CBC) is widely used in screening for haematological diseases, and most CBCs can be completed within 1 hour (3). APL can be identified by a particular abnormal promyelocyte morphology characterised by kidney-shaped or bilobed nuclei and a heavily granulated cytoplasm (1). Once abnormal promyelocytes are found, ATRA could be applied to reduce the risk of fatal bleeding. Therefore, microscopic morphological assessment of blood smears is a fast and effective way for early screening of APL (7, 10).

However, despite the speed and effectiveness of microscopic morphological assessment of blood smears, it has several shortcomings, including interobserver variability and difficulty in training suitable personnel (10–13). These shortcomings have led to an urgent need to develop an effective way to identify abnormal promyelocytes and improve the effectiveness of early screening of APL.

In recent years, significant progress has been made in using artificial intelligence (AI) in image recognition and processing and its implementation in clinical practice (14–16). The application of a fully automatic digital cell morphology analyser could significantly shorten the turnaround time (TAT) for morphology assessment and improve efficiency (14, 17, 18). Previous studies have investigated the use of AI to diagnose APL in bone marrow smears (7, 14). Compared to other automated digital morphology analysers, MC-100i can identify abnormal promyelocytes in blood smears, which is innovative and convenient in APL screening. To our knowledge, this is the first study evaluating the performance of MC-100i, a new digital cell morphology analyser, in identifying abnormal promyelocytes in blood smears and assisting in the early screening of APL. We found that the MC-100i classification results were precise, accurate and consistent with the manual classification results. MC-100i had excellent sensitivity and specificity for the early screening of APL. Moreover, MC-100i also improved the TAT for morphology assessment.

## 2 Materials and methods

### 2.1 Patients

A total of 192 patients admitted to the Emergency Department of the First Affiliated Hospital of Sun Yat-sen University between July 2023 and January 2024 were enrolled. All the enrolled patients had bleeding symptoms and were required to undergo a CBC by a physician in the emergency room. Another 49 patients with bleeding symptoms were excluded because they were diagnosed with leukaemia before. Among the 192 patients, 46 were subsequently diagnosed with APL, 96 were diagnosed with haematological malignancies other than APL, and 50 were diagnosed with bleeding disorders with normal white blood cell (WBC) morphology, including 35 with immune thrombocytopenic purpura (ITP), 5 with disseminated intravascular coagulation (DIC), 5 with thrombotic microangiopathy, 3 with haemophilia A and 2 with liver cancer. According to the revised fifth edition of the WHO Classification of Tumours of Haematopoietic and Lymphoid Tissues (8), the diagnosis of APL was based on blood cell morphology, flow cytometry and the presence of the *PML*::*RARA* fusion gene, as detected by both fluorescence *in situ* hybridisation (FISH) and polymerase chain reaction (PCR). The median age of the 192 patients was 37 years (7–68 years), with 117 males and 75 females. All the APL patients had the *PML*::*RARA* fusion gene. Leukopenia occurs most frequently in APL (18/50), anaemia and thrombocytopenia are common features of all leukaemia cases.

The detailed characteristics of the patients are shown in **Table 1**.

**Table 1:** Characteristics of 192 patients suspected of having APL.

	APL	Haematological malignancy	Thrombocytopenia
n	46	96	50
Median age (range)	37 (2,84)	46 (2,83)	52.5 (10M, 82)
Sex			
Male	22	72	25
Female	24	24	25
Acute leukaemia type (n)			
AML	/	58	/
ALL	/	34	/
MDS	/	4	/
Leukopenia (<2.0×10^9^/L) (n) %	18 36.00%	6 6.25%	10 20.00%
WBC (10^9^/L) median(IQR)	5.86 (1.19,16.75)	40.61 (10.41,94.02)	4.33 (2.45,6.85)
HGB (g/L) median(IQR)	71.5 (60.5,89.5)	76 (64.75,95)	83.5 (75.25,108.5)
PLT count (10^9^/L) median(IQR)	34.5 (15.75,49)	40 (31,62)	49.5 (35.5-62)

APL, acute promyelocytic leukaemia; AML, acute myelocytic leukaemia; ALL, acute lymphocytic leukaemia; MDS, myelodysplastic syndrome.

We employed a prospective method to collect samples and smears. For each patient, two millilitres of blood were collected in the EDTA-K_2_ anticoagulant tube. CBCs were measured via a BC-6800 Plus automatic hematology analyser (Mindray, Shenzhen, China). Blood smears were generated and dyed using the fully automatic SC-120 slide maker (Mindray, Shenzhen, China). All the slides were then scanned and analysed digitally via a fully automated blood cell morphology analyser MC-100i (Mindray, Shenzhen, China) for automatic preclassification.

The Medical Ethics Committee of the First Affiliated Hospital of Sun Yat-sen University approved this study (Approval number [2023]462). Informed consent was not required because only residual samples were collected from the patients and tested.

### 2.2 MC-100i automated digital blood cell morphology analyser

In our study, to ensure standardised thickness and staining, all blood smears are generated and dyed by SC-120 and transported to MC-100i on an assembly line automatically. MC-100i scans the blood smears automatically with a microscope photography system to locate and capture high-definition and colourful blood cell images and analyzes the morphology of the WBCs, RBCs, and PLTs.

The analyser first uses low-magnification objective lens to scan smears, locate and determine the area suitable for microscopic examination, which is defined as the monolayer region with evenly distributed cells, avoiding edges or clumped areas. Then the analyser photographs the area under the low-magnification objective lens, to locate and identify the target cells. After that, the analyser switches to use the high-magnification objective lens and photographs the target cells or areas based on the position information obtained by the low-magnification objective lens (18, 19).

The MC-100i collects cell images from smears for preclassification (18, 20), which is defined as the collection of two hundred WBCs on each smear by MC-100i. MC-100i scans the monolayer area of the blood smear and divides the cells into separate images with a resolution of not less than 160,000 pixels (400×400), allowing the analysis of WBCs, red blood cells (RBCs) and platelets (**Supplementary Figure S1**). The analyser extracts from the captured cell images the characteristics of colours, texture and geometry, so as to obtain the “eigenvector” that represents the cell classes. Then the analyser assigns a class to each cell based on the information of the eigenvector (20).

**Figure 1:**
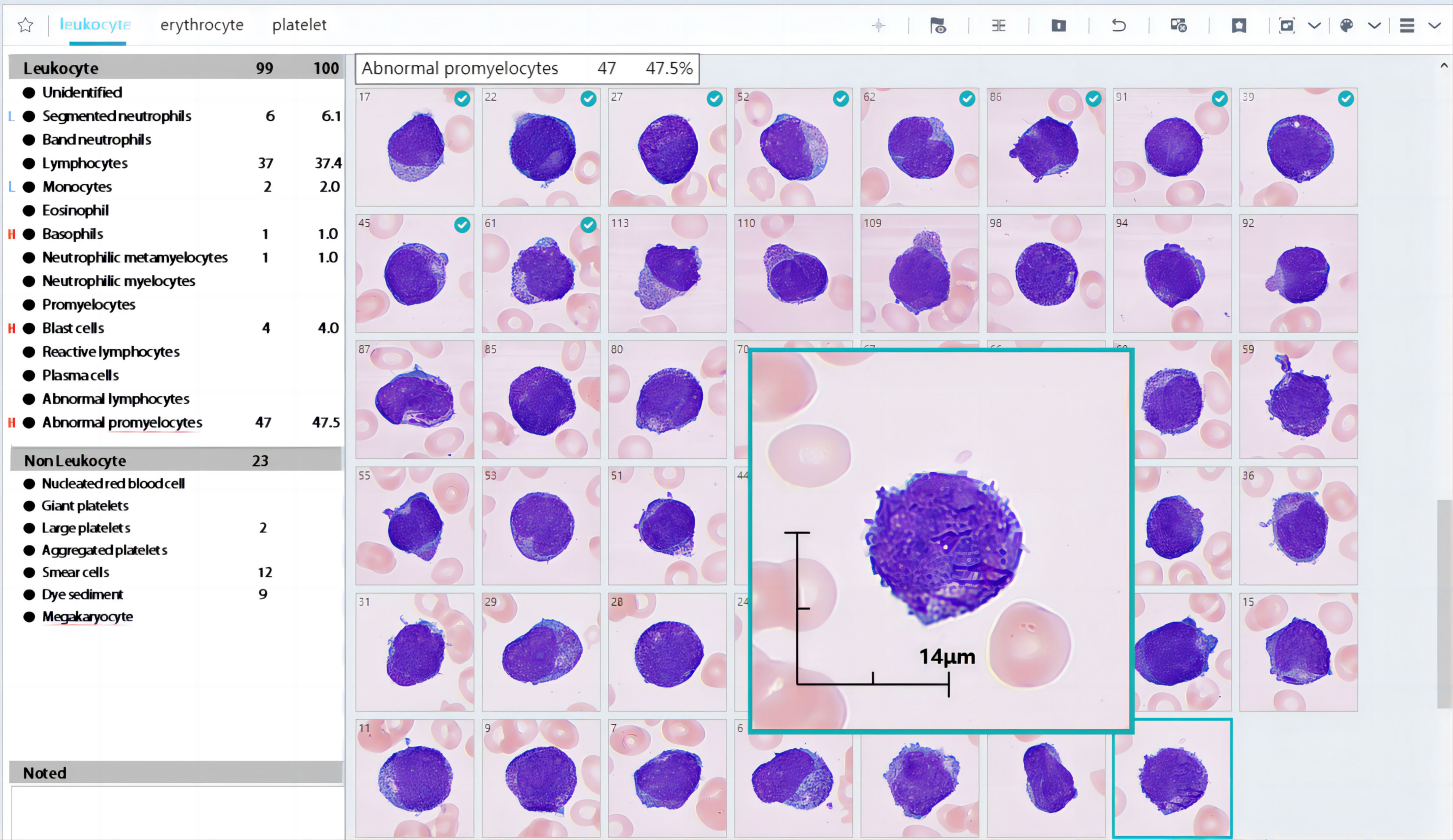
Partial results of MC-100i. The preclassification identified many abnormal promyelocytes in a blood smear. The cells with a green tick in the left-right corner were reclassified by the technologist.

In our study, we analysed only the WBC classification mode. MC-100i can preclassify WBCs into 15 types, including segmented neutrophils and band neutrophils, lymphocytes, monocytes, eosinophils, basophils, myelocytes, metamyelocytes, promyelocytes, blasts, reactive lymphocytes, plasma cells, abnormal promyelocytes and abnormal lymphocytes (both for research uses only) and unidentified cells. Blood cell images can be displayed with LabXpert software (Mindray, Shenzhen, China) and subsequently reclassified by technologists (**Figure 1**). The results after reclassification by technologists are defined as the postclassification results in the remainder of the manuscript.

### 2.3 Precision study

A precision study was conducted to evaluate the repeatability of MC-100i in preclassifying WBCs according to guidelines previously published by the International Council for Standardisation in Haematology (ICSH) (21, 22). Two hundred WBCs were set to be counted on each preclassification. Ten repeated WBC preclassifications were performed with MC-100i on 20 APL blood smears, 20 acute leukaemia blood smears and 20 thrombocytopenia blood smears. The mean, standard deviation (SD) and inaccuracy (CV) for each cell type were calculated. According to the literature and the manufacturer’s suggestion, the acceptable standard is an SD less than or equal to 2.5 (17).

### 2.4 Accuracy study

The sensitivity and specificity of MC-100i for each cell type were calculated to assess the preclassification performance. Blood smears from all 192 patients were included. Two hundred WBCs were preclassified on each slide by MC-100i, which was defined as the preclassification result. The preclassified cell images were then reclassified and validated by a morphology expert with more than 15 years of blood cell morphology assessment experience, defined as the postclassification results, which were then confirmed by another morphology expert with 20 years of experience in blood cell morphology assessment. When the preclassification result is inconsistent with the postclassification result, the latter shall prevail. If discrepancies exist between the morphology experts, another morphology expert with 20 years of was consulted and the postclassification result would be discussed and determined by the three morphology experts. The sensitivity and specificity of MC-100i for each cell type were then calculated using the postclassification results as the reference.

### 2.5 Consistency study

To assess the ability of MC-100i to identify abnormal promyelocytes, Passing-Bablok regression and Bland-Altman plot analyses were performed to compare the percentages of abnormal promyelocytes in the preclassification, postclassification and manual classification results. Two morphology experts classified the WBCs in the 46 blood smears with a microscope (200 WBCs on each smear). The mean percentage of abnormal promyelocytes in a smear was recorded as the manual count result if the difference in the percentage of abnormal promyelocytes between the two morphologists was less than 10%. Another morphology expert with 20 years of expertise confirmed the results through microscopy if the difference in the percentage of abnormal promyelocytes between the first two experts was greater than 10%. Clinical manifestations, preclassification and postclassification results were not provided to the morphologists (18).

Passing-Bablok regression is a nonparametric statistical analysis technique commonly used for methodological comparative studies in medical diagnostic research. Suppose that the confidence interval(CI) of the population intercept (α) does not include 0 or that the confidence interval of the population regression coefficient (slope) (β) does not include 1; this suggests the presence of systematic differences or proportional differences between the two methods (19). Bland-Altman analysis, which employs a two-dimensional Cartesian plot, assesses the consistency in differences between two variable groups by requiring that 95% of the data points fall within the 95% confidence interval (95%CI) of the limits of agreement (LoA), but it is required that the 95%CI of the mean of the difference includes or is close to 0 (20).

### 2.6 Clinical sensitivity and specificity

Clinical sensitivity and specificity were calculated to assess the clinical practicality of MC-100i in assisting with the early screening of APL. The data from all 192 patients were included in the calculation of clinical sensitivity and specificity. Clinical sensitivity was calculated by dividing the number of patients correctly preclassified as having abnormal promyelocytes and subsequently diagnosed with APL by the total number of patients preclassified as having abnormal promyelocytes by MC-100i. Clinical specificity was calculated by dividing the number of patients correctly preclassified as not having abnormal promyelocytes and subsequently diagnosed as not having APL by the total number of patients preclassified as not having abnormal promyelocytes by the MC-100i.

### 2.7 Turnaround time comparison

To compare the efficiency between classification with MC-100i and classification with manual microscopy, the mean turnaround time (TAT) of 2 technologists, with 3 and 15 years of blood cell morphology experience, was calculated and recorded, respectively. All 192 smears were involved. The processes of MC-100i classification and manual microscopic classification were divided and defined as follows:

Slide inserted into the instrument: the process of inserting a slide into MC-100i;
Preclassification: the process of MC-100i classification of 100 WBCs in a blood smear;
Postclassification: the process in which the technologist checks the preclassification results and makes modifications;
Result recording: the process of recording the classification results into the laboratory information system;
Slide placement onto the microscope stage: the process of placing the slide on the microscope stage before microscopic observation;
Ideal area scanned under a low-power field (100x): the process of scanning an ideal area for high-power observation with a low-power lens;
Cell count under high power (1000x): the process of differentiating 200 WBCs in the microscope with a high-power lens.

As the WBC count and the presence of blast cells (including blasts and abnormal promyelocytes) could affect the classification time, the blood smears were grouped according to whether blast cells were present in the manual classification and whether the WBC count in the automatic haematology analyser was lower than 2.0×10^9^/L, respectively.

### 2.8 Statistical and data analysis

The demographic data are presented as medians and ranges. Precision data are presented as the means ± standard deviations. The precision and accuracy studies were performed with Microsoft Excel (version 2016). Passing-Bablok and Bland-Altman analyses were performed with MedCalc statistical software (version 20). *P* values <0.05 were considered to indicate statistical significance.

## 3 Results

### 3.1 Excellent precision in the MC-100i preclassification results

To assess the precision of the MC-100i preclassification results, ten repeated WBC preclassifications were performed on sixty blood smears. The SDs for all the cell types were less than 2.0 (**Table 2**). MC-100i achieved excellent precision in terms of its preclassification results including abnormal promyelocytes.

**Table 2:** Results of the precision study of MC-100i.

Cell class	Average percentage of cells per slide, median (IQR)	SD, median (IQR)	%CV, median (IQR)
Blasts	21.9 (3.0-66.5)	1.5 (1.0-2.5)	10.0 (3.4-22.6)
Abnormal promyelocytes	24.6 (1.8-31.9)	1.7 (0.9-3.1)	10.4 (8.1-28.5)
Promyelocytes	9.9 (1.2-18.1)	1.1 (0.4-2.4)	12.1 (6.8-30.6)
Neutrophilic myelocytes	2.3 (1.0-5.6)	0.7 (0.5-1.3)	21.4 (4.3-49.9)
Neutrophilic metamyelocytes	1.6 (1.0-3.6)	0.5 (0.0-0.9)	22.5 (0.0-32.2)
Band neutrophils	3.2 (1.2-14.5)	0.8 (0.4-1.5)	18.0 (6.9-27.2)
Segmented neutrophils	11.9 (3.8-78.7)	1.4 (0.7-2.0)	5.2 (2.0-21.4)
Eosinophils	2.4 (1.0-7.2)	0.8 (0.4-1.1)	21.1 (9.9-31.1)
Basophils	1.9 (1.0-4.0)	0.5 (0.0-0.9)	18.0 (0.0-27.6)
Lymphocytes	27.4 (15.1-53.5)	1.7 (1.0-2.3)	5.9 (3.2-10.6)
Monocytes	6.5 (2.3-11.9)	1.0 (0.7-1.7)	18.9 (8.2-31.6)
Reactive lymphocytes	1.0 (0.9-1.3)	0.6 (0.5-0.7)	47.1 (35.1-63.1)
Nucleated red blood cells	1.8 (1.0-2.6)	0.6 (0.1-0.7)	19.9 (0.0-25.9)

### 3.2 MC-100i showed perfect accuracy in the comparison between preclassification and postclassification results

To assess the accuracy of MC-100i preclassification results, the preclassification and postclassification results of 37930 cells from 192 blood smears were analysed. MC-100i had excellent performance in preclassifying normal cell types, including neutrophilic metamyelocytes, band neutrophils, segmented neutrophils, eosinophils, basophils, lymphocytes and monocytes, with sensitivity and specificity values all above 95%. MC-100i can identify most blood cells with high accuracy. However, while the specificities for blasts, abnormal promyelocytes, promyelocytes and neutrophilic myelocytes were all above 97%, the respective sensitivities were relatively low. The sensitivity of abnormal promyelocytes was only 62.8%. Among the 5260 abnormal promyelocytes, 563 were preclassified as promyelocytes, 519 were preclassified as monocytes, 465 were preclassified as blasts, 211 were preclassified as myelocytes and 162 were preclassified as metamyelocytes (**Table 3**). These misclassifications all occurred in the cell types that are morphologically similar to abnormal promyelocytes.

**Table 3:** Preclassification performance of MC-100i.

		Preclassification															
		Blast	AP	Pm	Mye	Meta	Nst	Nsg	Eos	Baso	Lym	Mono	PC	RL	AL	U	Total
Post- classifi- cation	Blast	10261	73	222	8	4	0	0	4	22	44	59	20	67	23	0	10807
	AP	465	3301	563	211	162	0	5	13	12	0	519	0	0	9	0	5260
	Pm	12	13	282	7	0	0	0	0	0	0	0	0	0	20	0	334
	Mye	4	5	16	314	0	0	0	0	0	0	0	0	0	0	0	339
	Meta	2	0	0	5	318	0	0	0	0	0	0	0	0	0	0	325
	Nst	0	0	0	0	9	1587	14	0	0	0	0	0	0	0	0	1610
	Nsg	0	0	0	0	0	33	8559	2	60	0	2	0	0	0	0	8656
	Eos	0	0	0	2	2	0	4	347	0	0	0	0	0	2	0	357
	Baso	0	0	0	0	0	0	3	0	261	0	0	0	0	5	0	269
	Lym	49	0	0	0	0	0	0	0	0	8047	41	17	37	0	0	8191
	Mono	4	16	3	0	0	0	0	0	0	21	1570	0	16	8	0	1638
	PC	0	0	0	0	0	0	0	0	0	0	0	0	0	0	0	0
	RL	4	0	0	0	0	0	0	0	0	21	4	21	77	17	0	144
	AL	0	0	0	0	0	0	0	0	0	0	0	0	0	0	0	0
	U	0	0	0	0	0	0	0	0	0	0	0	0	0	0	0	0
	Total	10801	3408	1086	547	495	1620	8585	366	355	8133	2195	58	197	84	0	37930
True Positives		10261	3301	282	314	318	1587	8559	347	261	8047	1570	0	77	0		
False Negatives		546	1959	52	25	7	23	97	10	8	144	68	0	67	0		
True Negatives		26583	32563	36792	37358	37428	36287	29248	37554	37567	29653	35667	37872	37666	37846		
False Positives		540	107	804	233	177	33	26	19	94	86	625	58	120	84		
Sensitivity		94.9%	62.8%	84.4%	92.6%	97.8%	98.6%	98.9%	97.2%	97.0%	98.2%	95.8%	0.0%	53.5%	0.0%		
Specificity		98.0%	99.7%	97.9%	99.4%	99.5%	99.9%	99.9%	99.9%	99.8%	99.7%	98.3%	99.8%	99.7%	99.8%		

Blast, Blasts; AP, Abnormal promyelocytes; Pm, Promyelocytes; Mye, Neutrophilic myelocyte; Meta, Neutrophilic metamyelocytes; Nst, Band neutrophils.

Nsg, Segmented neutrophils; Eos, Eosinophils; Baso, Basophils; Lym, Lymphocytes; Mono, Monocytes; PC, Plasma cells; RL, Reactive lymphocytes; AL, Abnormal lymphocytes.

U, Unidentified. Yellow shadings emphasize the numbers of correctly preclassified cells.

### 3.3 The MC-100i classification results were consistent with the manual classification results

**Figure 2:**
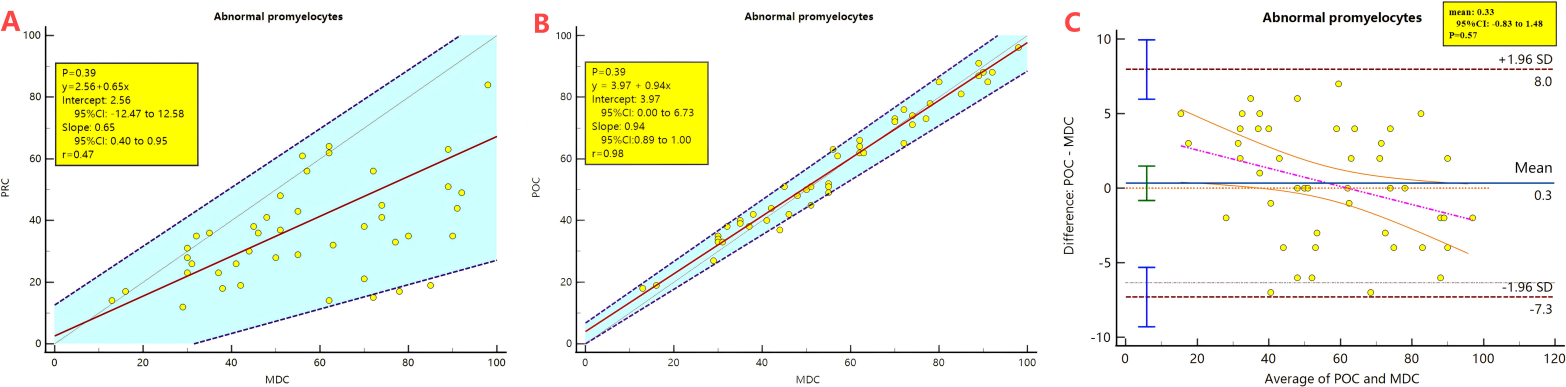
Passing-Bablok regression and Bland-Altman plot analysis comparing the preclassification and the postclassification with manual classification results of abnormal promyelocyte percentages. PRC, Preclassification; POC, postclassification; MC, manual classification. **(A)** Passing-Bablok regression analysis between the preclassification and manual classification. **(B)** Passing-Bablok regression analysis between the postclassification and manual classification. **(C)** Bland-Altman plot analysis analysis between the postclassification and manual classification.

Manual classification found abnormal promyelocytes in all 46 APL smears. The percentage of abnormal promyelocytes ranged 1- 84% on blood smears. After analysing by the Passing-Bablok regression there is a proportional difference between the MC-100i preclassification and manual classification results (the 95% CI of the slope does not include 1). The correlation coefficient r=0.47, which indicates that the correlation coefficient between the two methods is relatively low (0.30-0.50) (**Figure 2A**).

The 95%CI of the mean of the difference between the two methods is -26.55 to -14.75, which is significantly below 0, the Bland-Altman analysis is not applicable.

After the morphologist reclassified the abnormal promyelocytes, Passing-Bablok regression analysis revealed that the postclassification and manual classification results had no systematic or proportional differences. The correlation coefficient r=0.98 indicates that the correlation coefficient between the two methods is very high (>0.90) (**Figure 2B**). The 95% CI of the mean of the difference is -0.83 to 1.48 containing 0, Therefore, the Bland-Altman can be applied for analysis. The Bland-Atman plot revealed that the postclassification results were relatively consistent with the manual classification results. (**Figure 2C**)

### 3.4 MC-100i had high clinical sensitivity and specificity in the early screening of APL

As the MC-100i classification results were consistent with the manual classification results, its clinical sensitivity and specificity were further studied. The clinical sensitivity and specificity of MC-100i preclassification for the early screening of APL were 100.0% and 98.6%, respectively, whereas those of postclassification and manual classification for the early screening of APL were both 100% (**Table 4**). MC-100i had high clinical sensitivity and specificity in the early screening of APL.

**Table 4:** Clinical sensitivity and specificity in the early screening of APL.

Patients for whom abnormal promyelocytes could be identified	APL (n=46)	Acute Leukaemia (n=96)	Thrombocyt- openia (n=50)	Clinical sensitivity for APL	Clinical specificity for APL
Preclassification	46	2	0	100.0%	98.6%
Postclassification	46	0	0	100.0%	100.0%
Manual classification	46	0	0	100.0%	100.0%

### 3.5 Turnaround time comparison

To assess the efficiency of using MC-100i, processes of MC-100i classification and manual microscopy classification were divided and recorded. The TAT records were then analysed. For both the expert and the technologist, the mean TAT with the MC-100i was significantly shorter than the mean TAT with manual classification. The technologist’s postclassification time and cell-counting time were significantly longer than those of the expert (*P*<0.01). However, the mean TAT of the technologist with MC-100i was significantly shorter than the mean TAT of the expert with manual classification (*P*<0.01).

**Figure 3:**
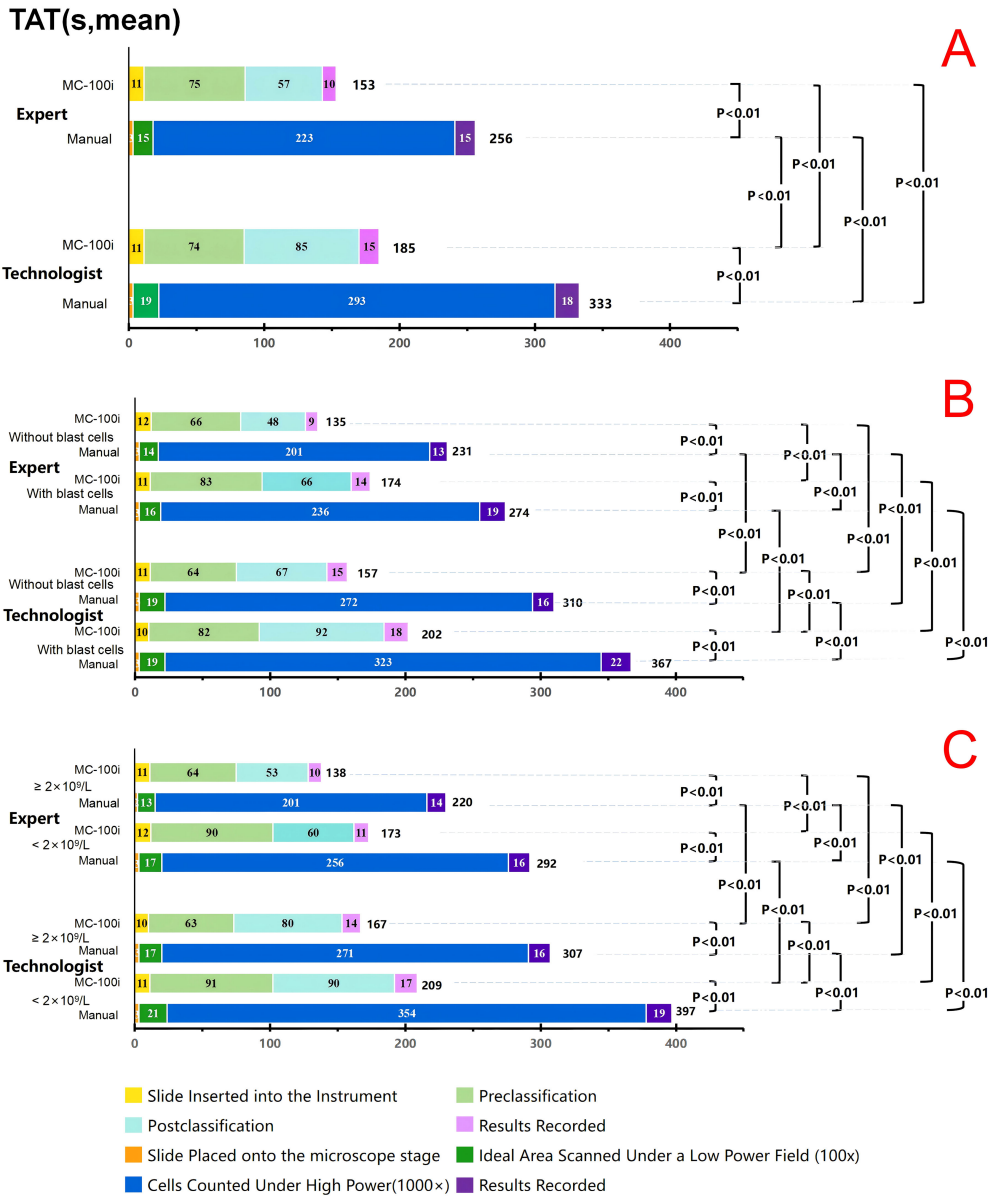
Turnaround time (TAT) for the expert and the technologist. **(A)** TAT comparison between the expert and technologist. **(B)** TAT comparison between the expert and technologist in blood smears with and without blast cells (including blasts and promyelocytes). **(C)** TAT comparison between the expert and technologist in blood smears with WBC ≥2×10^9^/L and <2×10^9^/L. Expert: Technologist with more than 15 years of morphology experience. Technologist: Technologist with 3 years of morphology experience. MC-100i: TAT with MC-100i Manual: TAT with manual classification Without blasts: TAT for blood smears without blast cells. With blasts: TAT for blood smears with blast cells. ≥2×10^9^/L: TAT for blood smears with a WBC count not lower than 2×10^9^/L <2×10^9^/L: TAT for blood smears with a WBC count lower than 2×10^9^/L.

For both the expert and the technologist, both the mean TAT and the manual classification time were longer if blast cells were present or if the WBC count was low; however, the mean TAT was shortened with the use of MC-100i. Moreover, the mean TAT of the technologist with MC-100i was significantly shorter than the mean TAT of the expert with manual classification (*P*<0.01) when blast cells or a low WBC was present. For both the expert and the technologist, the postclassification time with MC-100i was significantly longer when blast cells or a low WBC was present. (*P*<0.01) (**Figure 3**).

## 4 Discussion

In this study, we assessed the performance of MC-100i, a new digital cell morphology analyser, in identifying abnormal promyelocytes in blood smears and assisting in the early screening of APL. We found that the MC-100i classification results were precise, accurate and consistent with the manual classification results. MC-100i had excellent sensitivity and specificity for the early screening of APL. It also improved the TAT for morphology assessment.

The identification of abnormal promyelocytes in blood smears is important for the early screening of APL (9, 23). However, the identification of abnormal promyelocytes currently relies on manual microscopic assessment, which is limited by long time consumption, interobserver variability, and, most importantly, difficulty in training personnel (10–13). Therefore, the current guidelines recommend exploring a faster and more effective method for the early screening of APL (10, 24).

Eckardt et al. developed a multistage deep learning platform that automatically reads images of bone marrow smears, accurately segments cells, and subsequently predicts APL using image data (7). Ouyang et al. developed a CNN-based approach for diagnosing APL in bone marrow images (14). Manescu et al. developed a deep learning-based approach that can detect acute promyelocytic leukaemia (25). However, these studies used images of the bone marrow smears, which are acquired by invasive bone marrow acupuncture. Treatment for APL is time-sensitive. Early ATRA administration could reduce the risk of fatal bleeding and cure most APL patients (2, 3). Therefore, rapid and early screening is crucial to reduce the early death rate in APL patients (7). To our knowledge, this is the first study to evaluate the effectiveness of MC-100i, which is an automated device for identifying abnormal promyelocytes in blood smears and assisting in the early screening of APL.

In the precision study, the performance of MC-100i was assessed with blood smears with normal and abnormal WBC classification. MC-100i showed excellent precision, with median SDs for all cell types less than 2.0, suggesting comparable performance with other automated digital blood cell morphology analysers (18, 19, 25–27). The median SDs for blasts and abnormal promyelocytes were greater than those for other cell types but still less than 2.0. These findings suggest that MC-100i can be used to preclassify WBCs and identify blasts and abnormal promyelocytes with repeatable and stable performance. Excellent precision is the basis of its application in the early screening of APL.

The literature has also shown that other automated digital blood cell morphology analysers (Cellavision DM96, Cellavision DI-60, Mindray MC-80) performed well in identifying most WBC types, but their performance decreased when blast cells (including blasts and abnormal promyelocytes) are present (19, 27–30). In our accuracy study, the preclassification results indicated that MC-100i had excellent performance in identifying most WBC types. However, the sensitivities for blasts, abnormal promyelocytes and promyelocytes were relatively low. These findings suggest that MC-100i can identify normal blood cells with high accuracy, but its performance could be affected by the presence of blasts and abnormal promyelocytes (1). MC-100i tends to misclassify certain atypical abnormal promyelocytes with less granularity as blasts or monocytes. These atypical cells are highly variant and difficult to classify, even by some laboratory technologists. It should be emphasised that when reviewing the preclassification results, morphologists should be aware that abnormal promyelocytes could be misclassified as promyelocytes, monocytes, blasts, myelocytes, and metamyelocytes. It could be inferred that MC-100i can be used as an effective tool for screening APL. Even if some abnormal promyelocytes may be misclassified by MC-100i, they could be reclassified by technologists.

In the consistency study, the MC-100i preclassification results for abnormal promyelocytes correlated well with the manual classification results. However, Passing-Bablok regression revealed that the preclassification results of MC-100i were proportionally different from the manual classification results, which is consistent with similar evaluations in the literature (17–19, 29, 31). After the morphologists’ reclassification, there were no systematic or proportional differences between the postclassification results for abnormal promyelocytes and the manual classification results. Bland-Altman analysis revealed that the difference between the postclassification and manual classification results was within the limits of agreement, suggesting that MC-100i postclassification had similar effectiveness to manual classification in identifying abnormal promyelocytes. However, reclassification is necessary to obtain an accurate percentage of abnormal promyelocytes. MC-100i could be useful for identifying abnormal promyelocytes and early screening of APL. However, further efforts could be made to improve the accuracy of MC-100i preclassification in abnormal promyelocytes.

Although misclassification occurs in preclassification, for APL early screening, correctly identifying abnormal promyelocytes is more crucial than accurately counting the percentage of abnormal promyelocytes in the blood smears. APL should be suspected if any abnormal promyelocyte is identified; in such circumstances, ATRA should be administered regardless of the accurate percentage of abnormal promyelocytes present in the smears. In our study, MC-100i identified abnormal promyelocytes in all the APL blood smears. Although it misidentified some cells as abnormal promyelocytes, the clinical sensitivity of MC-100i in the early screening of APL was 100.0%, and the clinical specificity was 98.6%, which was very close to the performance of the postclassification and manual classification results. For clinical application, on the one hand, misclassifications in the preclassification can be considered tolerable so long as MC-100i is sensitive in identifying abnormal promyelocytes; on the other hand, when abnormal promyelocytes are identified, to report accurate WBC classification results, morphologists still need to scrutinise the morphology of all cell types and make necessary reclassifications. These findings suggest that MC-100i could effectively assist in the early screening of APL.

The TAT is an important tool for analysing the effectiveness of an automated digital blood cell morphology analyser. Lee et al. reported comparable TATs between the Cellavision DC-1 digital blood cell morphology analyser and manual classification (26). Nam et al. found that the TAT of a Cellavision DI-60 digital blood cell morphology analyser was longer than the TAT of manual classification (32). The TAT analysis in the literature did not show an advantage in using automated digital morphology analysers. To analyse the TAT, we recorded the detailed time spent on classification with MC-100i and with microscopy. The results showed that the use of MC-100i significantly shortened the classification TAT for both the technologist and the expert. In particular, for blood smears with blast cells and low WBC counts, MC-100i significantly shortened the classification TAT. Furthermore, the technologist’s classification time with MC-100i was significantly shorter than the expert’s classification time with a microscope. Our findings indicate that MC-100i could effectively assist technologists in blood cell classification, especially for blood smears with blast cells and low WBC counts. MC-100i could also be very effective in assisting in the early screening of APL when experienced technologists are not available. It can be also advantageous when confirmation of t (15;17) by cytogenetics or *PML*::*RARA* by FISH or PCR is still pending and in low-resource settings where such advanced tests are not available (7).

There are several limitations in our study. First, this was a single-centre study with a relatively small number of cases. Second, as the incidence of variant APL is relatively low (about 5% in APL cases) (33), we did not encounter any variant APL during our study, which may affect the accuracy of the morphological judgement of MC-100i. Although AML with *PML*::*RARA* represents about 95% of APL cases in clinical practice, to further study the generalisability of MC-100i in the early screening of APL, a multicentre study with more APL cases and more variant APL types could be considered for future research.

## 5 Conclusions

Our findings indicate that MC-100i is an effective tool for identifying abnormal promyelocytes in blood smears. MC-100i can be used to assist in the early screening of APL. It is useful when experienced morphological experts or advanced tests are not available. Future work will focus on increasing sample size and multicentre validation of MC-100i’s feasibility.

## Data Availability

The raw data supporting the conclusions of this article will be made available by the authors, without undue reservation.

## Supplementary material

Supplementary Figure 1 Cell classification with convolutional neural networks (CNNs) in MC-100i. CNN employs deep learning methods and can recognise blood cell images accurately. Through multilevel and multichannel convolution templates, CNNs automatically extract image features and progressively decompose them from low-dimensional to high-dimensional features. These high-dimensional features contain information that cannot be perceived by the human eye. The CNNs then further integrate and analyse these features, establishing correlations between cell types and multidimensional cell features. This enables the CNNs to self-learn and thus achieve better performance in classifying cells.
