# Supplementary material for: Artificial intelligence-assisted early screening of acute promyelocytic leukaemia in blood smears: a prospective evaluation of MC-100i
Source: Front Oncol. 2025 Apr 7;15:1572838. doi: 10.3389/fonc.2025.1572838 (PMC12010105; doi:10.3389/fonc.2025.1572838)
Supplement: Supplementary Figure 1 — Cell classification with convolutional neural networks (CNNs) in MC-100i. CNN employs deep learning methods and can recognise blood cell images accurately. Through multilevel and multichannel convolution templates, CNNs automatically extract image features and progressively decompose them from low-dimensional to high-dimensional features. These high-dimensional features contain information that cannot be perceived by the human eye. The CNNs then further integrate and analyse these features, establishing correlations between cell types and multidimensional cell features. This enables the CNNs to self-learn and thus achieve better performance in classifying cells. [file SupplementaryFile1.docx]

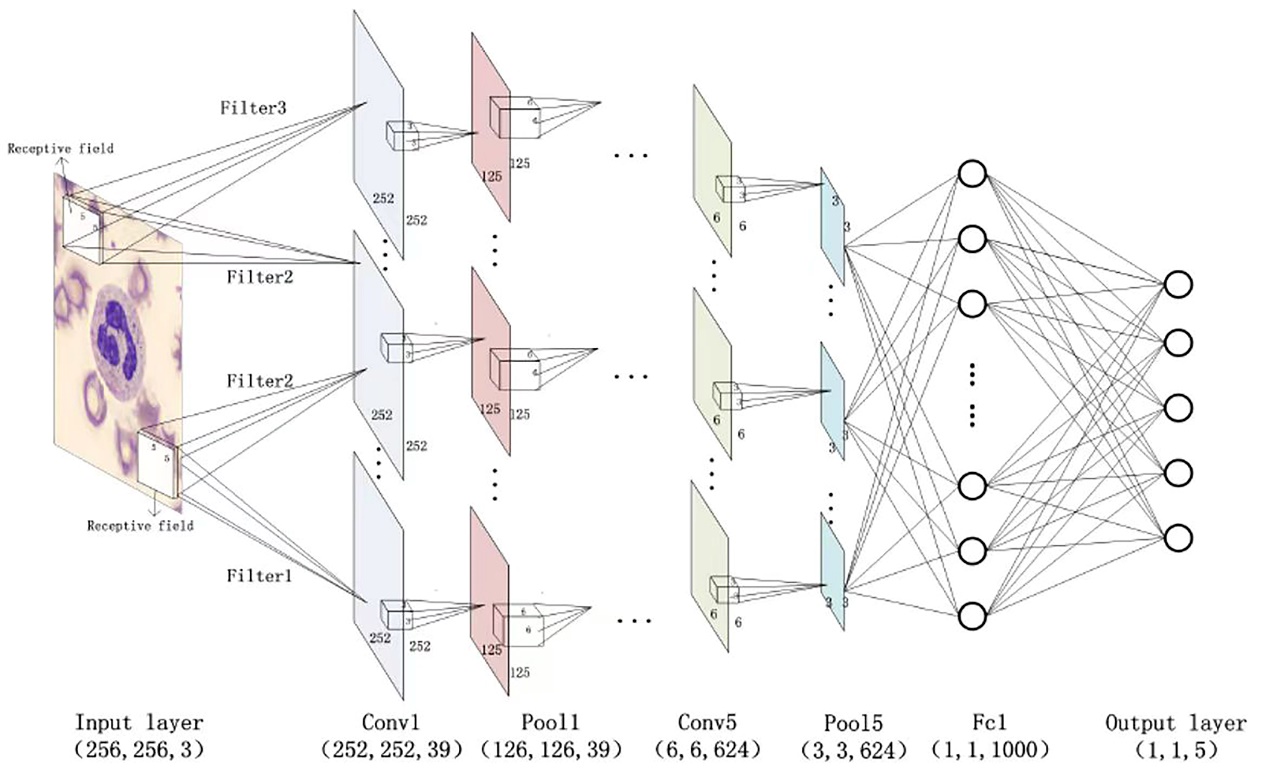


Figure S1 Blood cell classification with convolutional neural networks (CNNs) in MC-100i. CNN employs deep learning methods and can recognise blood cell images accurately. Through multilevel and multichannel convolution templates, CNNs automatically extract image features and progressively decompose them from low-dimensional to high-dimensional features. These high-dimensional features contain information that cannot be perceived by the human eye. The CNNs then further integrate and analyse these features, establishing correlations between cell types and multidimensional cell features. This enables the CNNs to self-learn and thus achieve better performance in classifying cells.
